# Respiratory Disease Risk of Zoo-Housed Bonobos Is Associated with Sex and Betweenness Centrality in the Proximity Network

**DOI:** 10.3390/ani11123597

**Published:** 2021-12-19

**Authors:** Jonas R. R. Torfs, Marcel Eens, Daan W. Laméris, Nicky Staes

**Affiliations:** 1 Behavioral Ecology and Ecophysiology Group, Department of Biology, University of Antwerp, Universiteitsplein 1, 2610 Wilrijk, Belgium; marcel.eens@uantwerpen.be (M.E.); daan.lameris@uantwerpen.be (D.W.L.); nicky.staes@uantwerpen.be (N.S.); 2 Centre for Research and Conservation, Royal Zoological Society of Antwerp, Koningin Astridplein 26, 2018 Antwerp, Belgium

**Keywords:** social-network analysis, social position, disease, welfare, captivity

## Abstract

**Simple Summary:**

One of the basic conditions of animal welfare is good health. While social behaviors have many benefits for group-living animals, they also play a role in disease transmission. This is especially true in primate species, like bonobos, who have complex social dynamics, which can facilitate disease transmission. Bonobos are also more susceptible to human disease variants due to their close genetic relatedness and are therefore at higher risk of infection in captivity due to closer proximity to humans. Therefore, investigation whether an individual’s characteristics, like sex, age, or social-network position increase disease risk provides information that can be used for future management decisions to improve general animal welfare. To do so, we monitored the occurrence of respiratory disease symptoms during one winter season in a relatively large group of 20 zoo-housed bonobos. We found that individuals that were more central in the social network had higher chances of contracting respiratory disease and that males were more likely to get infected than females. These results indicate that for bonobos, social behavior and sex influence the risk of contracting respiratory disease, two factors that can be taken into account when managing fission-fusion dynamics during disease outbreaks in this zoo-housed species.

**Abstract:**

Infectious diseases can be considered a threat to animal welfare and are commonly spread through both direct and indirect social interactions with conspecifics. This is especially true for species with complex social lives, like primates. While several studies have investigated the impact of sociality on disease risk in primates, only a handful have focused on respiratory disease, despite it being a major cause of morbidity and mortality in both wild and captive populations and thus an important threat to primate welfare. Therefore, we examined the role of social-network position on the occurrence of respiratory disease symptoms during one winter season in a relatively large group of 20 zoo-housed bonobos with managed fission-fusion dynamics. We found that within the proximity network, symptoms were more likely to occur in individuals with higher betweenness centrality, which are individuals that form bridges between different parts of the network. Symptoms were also more likely to occur in males than in females, independent of their social-network position. Taken together, these results highlight a combined role of close proximity and sex in increased risk of attracting respiratory disease, two factors that can be taken into account for further welfare management of the species.

## 1. Introduction

Group living is a widespread phenomenon across the animal kingdom, since it provides several benefits for an animal’s survival and reproductive success, such as reducing predation risk, increasing foraging efficiency, and providing easier access to mates [1,2,3,4]. However, a major cost of group living is increased exposure to parasites and pathogens due to close contact among group members [5,6,7]. Indeed, the prevalence of socially transmitted parasites and pathogens increases with group size [5,8], but heterogeneity in social behavior influences how disease-causing organisms spread throughout a population, thereby causing variation in infection risk for individual group members [5,8,9,10,11,12]. In other words, differences in social behavior among group members can influence the chance of an individual becoming infected with a pathogen, and this has, in turn, an important influence on the fitness and overall welfare of these individuals. Therefore, it is of great interest to identify which attributes of social behavior are responsible for disease transmission and susceptibility.

Individuals of the same group engage in non-random interactions with each other, such that individuals are embedded within a complex network of interactions among all group members [13,14,15,16]. Social-network analysis (SNA) provides a useful toolkit to study these complex networks and, by extension, the association between social behavior and infection risk. SNA can be used to construct social networks based on all types of social behavior, and from these networks, different measures can be calculated that represent an individual’s position in the network through its direct and indirect connections. It therefore informs us about which aspects of sociality are involved in the spread of disease through a population, or at what risk particular individuals are of becoming infected [17]. For example, it is expected that individuals with a high *degree* (i.e., the number of connections an individual has in a network) and *strength* (i.e., the total “activity” of an individual in the network, or in other words, the total weight of all its connections with other group members) in the network have higher infection risk since they come into contact with a large number of group members at a high rate [17]. This has been shown in a variety of species. For example, in gidgee skinks (*Egernia stokesii*), individuals with a higher *degree* in an association network had higher blood-parasite richness [18]. In meerkats (*Suricata suricatta*), a higher *degree* in a grooming network led to a higher risk of tuberculosis infection [19], and in Grant’s gazelles (*Nanger granti*), a higher *degree* in a group-membership network led to a higher risk of coccidia infection [20]. Finally, individuals with higher *strength* in association networks showed higher tick loads in pygmy bluetongue lizards (*Tiliqua adelaidensis*) [21] and higher infection risk with certain *Salmonella entereca* genotypes in Australian sleepy lizards (*Tiliqua rugosa*) [22].

However, these measures represent an individual’s direct connections in the network, and while they might be useful to investigate an individual’s immediate infection risk, they are less informative in terms of how diseases spread throughout the rest of the population. Indirect measures for connectivity are the *eigenvector centrality* (i.e., measure of an individual’s connectedness, as well as the connectedness of its associates [23]) and *betweenness centrality* (i.e., number of times an individual occurs on the shortest path between two other individuals in the network [24]). While individuals with high *eigenvector centrality* are strongly embedded within the network and therefore might be more prone to encounter socially transmitted pathogens [17], individuals with high *betweenness centrality* function as bridges between subgroups within the network, which increases their exposure to pathogens but also increases the risk of disease spread when these individuals are infected, as they are in contact with a larger proportion of the social network [17]. Therefore, taking indirect connections in the social network into account is vital to understand the link between social behavior and infection risk. Indeed, by simulating outbreaks of an infectious disease in a proximity network of free-ranging domestic dogs (*Canis familiaris*), it was found that when individuals with higher *eigenvector centrality* were infected first, larger outbreaks occurred, suggesting a vital role of these central individuals in disease outbreaks [25]. Moreover, in meerkats, higher *betweenness centrality* in an aggression network was associated with greater tuberculosis infection risk [19], and the same was found using proximity networks in badgers (*Meles meles*) [26] and common brushtail possums (*Trichosurus vulpecula*) [27].

Primates might be especially vulnerable to socially-spread diseases due to their complex social lives [28,29,30]. Indeed, some of the best-documented outbreaks of disease in animal populations have been found in primates (reviewed in [31]), such as outbreaks of Ebola [32,33,34,35,36], yellow fever [37,38], scabies [39], and various forms of respiratory disease [40,41,42,43,44,45,46,47,48]. Since these disease outbreaks can have a serious impact on the health and survival of both wild and captive populations, monitoring and understanding pathogen spread and the social factors influencing individual infection risk are therefore of high importance for the conservation and overall welfare of these species. Several studies have already investigated the association between individual social-network position and pathogen susceptibility in primates, using a variety of social behaviors. For example, brown spider monkeys (*Ateles hybridus*) that had a higher *degree*, *strength*, and *betweenness centrality* in the group’s contact network (i.e., a network based on all types of social contact behaviors) had higher overall gastrointestinal parasite richness and higher loads of *Strongyloides* and *Trychostrongylus* nematodes [49]. Similarly, *degree* and *betweenness centrality* in a contact network (i.e., a network based on grooming and huddling behavior) were positively correlated with *Escherichia coli* transmission in rhesus macaques (*Macaca mulatta*) [50], while *degree* and *eigenvector centrality* extracted from a grooming network were positively correlated with *Strongyloides* infection in female Japanese macaques (*Macaca fuscata*) [51]. In addition, *degree* and *betweenness centrality* predicted helminth infection in a proximity network of red-capped mangabeys (*Cercocebus torquatus*) [52]. Finally, *strength* in an association network based on same-subgroup membership predicted parasite richness in a group of chimpanzees (*Pan troglodytes*) [53]. Taken together, these studies appear to indicate a strong influence of individual differences in social-network position on parasite infection risk in primates.

While most research investigating infection risk and SNA in primates has focused on gastrointestinal parasites, other pathogens, such as viral respiratory diseases, remain understudied. Nevertheless, respiratory disease is a major cause of morbidity and mortality in both captive and wild primate populations and thus a prominent threat to general primate welfare [40,42,43,54,55,56]. To date, only two studies have investigated the link between SNA measures and the occurrence of respiratory disease in primates [57,58]. One study found no association between the social network and respiratory disease in mountain gorillas (*Gorilla beringei beringei*) [57], while another study showed that in chimpanzees, males with higher *strength* in a proximity network had a higher chance of showing symptoms of respiratory disease, indicating the vital role of the social-proximity network in the spread of the pathogen in the group [58]. However, this study focused solely on adult males and did not investigate the role of females and juveniles, which can also be important mediators of the spread of pathogens throughout the group [58].

Previous studies have indeed indicated that factors such as sex and age can influence disease risk. In many vertebrate taxa, females are observed to have fewer infections and stronger antibody responses than males [59,60,61,62,63,64]. Androgens, which occur in higher concentrations in males than females, can negatively affect the immune system [65,66,67]. Moreover, males might show behavior that increases their exposure risk, such as roaming in larger ranges than females or occupying other niches in the habitat [68]. In addition to sex, the age of an individual can also affect its infection risk. Across animal species, juveniles typically have higher infection incidence and intensity due to the lower efficiency of their immune system [51,58,69,70,71,72]. Moreover, as adults age, the strength of the immune system decreases, as has been shown by studies that found declines in markers of adaptive immunity or increases of inflammation with age in different vertebrate species [73,74,75,76,77]. As such, the relationship between age and disease susceptibility might be complex and U-shaped [78], with higher infection rates expected in young animals and elderly adults. Therefore, when investigating the risk of infection through social networks, the inclusion of all group members is needed to accurately map the spread of pathogens.

In this study, we investigate the association between individual characteristics, like sex, age, and position in the proximity network, on the occurrence of respiratory disease in a relatively large multi-male, multi-female group of 20 zoo-housed bonobos (*Pan paniscus*), with ages ranging from 0 to 43 years old. Outbreaks of respiratory disease are often seen in great apes, including bonobos [45,48,79]. Due to their close phylogenetic relatedness to humans, nonhuman great apes like bonobos are highly susceptible to human disease variants [80]. Hence, outbreaks of respiratory disease in great apes are often traced back to human contact, both in the wild [40,41,42,45,46,47,48,54,55,81,82,83] and in zoo-housed populations [84,85,86]. Due to the high prevalence of respiratory disease in bonobos and the risks it incurs for the welfare of the species, identifying the role of social behavior in viral transmission through social-network analysis can aid in identifying ways to contain outbreaks in the future.

We predicted that similar to what was previously described for male chimpanzees [58], the proximity network would be epidemiologically relevant in explaining the occurrence of respiratory disease symptoms in bonobos. We also predicted that *betweenness centrality* would be positively associated with the occurrence of respiratory disease symptoms. Given that no study to date has studied *betweenness centrality* in bonobos, we also investigated whether differences occur between the two sexes, with age, and between individuals that did or did not transfer between different subgroups in the population, given that the group is managed in a fission-fusion system. This will allow for interpretation of our results. We predict that individuals that transfer between groups will have higher *betweenness centralities* than individuals that remain in their subgroups, as they have more opportunities to form bridges between different parts of the subgroups. In addition, we also investigated the role of sex and age in determining disease risk. We hypothesized that males will have higher disease susceptibility than females and that disease susceptibility will be higher in young animals (<7 years old) and aged adults (>40 years old for bonobos [87]), compared to adults younger than 40 years old.

## 2. Materials and Methods

### 2.1. Data Collection and Study Sample

We conducted behavioral observations and health assessments of the bonobo group housed at Zoo Planckendael, Belgium between 14 January 2021 and 25 March 2021. This group consisted of 20 bonobos, 13 females and 7 males, between 14 days and 43 years old at the start of data collection (see Supplementary Table S1). During the study, the group was kept in two subgroups managed in a fission-fusion system, aiming to mirror fission-fusion dynamics found in wild bonobo populations [88,89]. This created large heterogeneity in social proximity, making this particular group an interesting study system to investigate the effect of social-network position on infection risk (cf. [58]). Due to the fission-fusion dynamics, these subgroups regularly changed composition. Certain dyads always remained together in the same subgroup, while 8 individuals regularly switched between subgroups in this fission-fusion system (see Supplementary Tables S1 and S2). Our dataset included four dependent infants (i.e., infants younger than 2 years old that spend a considerable amount of time on or in close proximity to their mothers) with limited mobility and thus similar proximity-network measures to their mothers, meaning they barely contribute to structuring the network itself. Nonetheless, these infants can be important mediators of pathogen spread due to the higher viral loads that they might carry [51,69,70,71,72], and therefore, they might be relevant to include in the analysis from an epidemiological perspective [90]. Therefore, we analyzed all data with and without these four infants.

Given that viral agents causing respiratory disease are typically airborne and can thus be transferred through time spent in close proximity [91], we chose to construct weighted social-proximity networks rather than using social-contact networks based on, for example, grooming, aggression, or play. Proximity data were collected using instantaneous group-scan sampling during daily observations [92]. Observations were done between 9:00 a.m. and 4:00 p.m. At 15 min intervals, a scan was done to determine the proximity of all individuals to their group members. Two individuals were recorded to be in proximity to each other when they were maximally 2 m (±two arm lengths of an adult bonobo) apart. In total, 1065 scans were done, with an average of 22 scans per day. Scans were recorded using a laptop with The Observer XT v14.0 software (Noldus, The Netherlands).

Health data were collected ad libitum by keepers during daily health assessments in the morning and by the researcher during observations throughout the day. Individuals that were noticed to cough multiple times per day or show rhinorrhea (i.e., nasal discharge, often colored) during the study period were considered to be infected with respiratory disease. Categorization of individual animals as “symptomatic” during the study period showed full agreement between keeper notes and researcher observations. Bonobos that never exhibited symptoms were considered to be healthy and non-infected during the study period (cf. [57,58]; see Supplementary Table S1).

### 2.2. Social Network Analysis

We created two adjacency matrices containing all pairwise proximity data: one containing all group members (*N* = 20) and one excluding the dependent infants (*N*_infants_ = 4). Since we focused the observations of one subgroup each day, not all dyads were observed equally. To correct for this, we divided the total amount of scans a dyad was seen in proximity by the total amount of scans that were done for this dyad. From this total amount of scans, we subtracted the number of scans where both individuals from a dyad were out of sight, since in these cases, it was unknown whether both dyad members were sitting in proximity. From this adjacency matrix, we constructed a weighted, undirected proximity-based social network and calculated *betweenness centrality* using the “ANTs” package [93] in R [94]. We chose to extract the *betweenness centrality* for each individual since it is considered the most relevant social-network measure for investigating patterns of disease spread in social networks [95]. This measure indicates to what extent an individual connects subgroups in the population and is therefore more or less likely to encounter pathogens as they are spread across the whole network [17]. We chose to focus on this measure only because different network metrics, like *strength*, *eigenvector centrality* and *betweenness centrality*, are often correlated [95,96], causing issues with collinearity, multiple comparisons, and interpretation [96].

### 2.3. Statistical Analyses

We used two methods to assess the relevance of the social network in infection risk. First, we assessed the relevance of the social network in the occurrence of symptoms, which we did for the full network and for the network without independent infants. We used a path-based k-test to assess whether the occurrence of symptoms of respiratory disease resulted from transmission of the disease along the network edges [97]. This test determines whether the mean distance between infected individuals in the social network is shorter than expected by chance. This is tested by “node-label swapping”. Cases of infection are randomly re-assigned within the network, and a null distribution of mean distances between infected individuals is created. We ran 1000 permutations. Then, a *p*-value is calculated as the proportion of random permutations in which the mean distance between infected individuals is smaller than the observed mean distance [97].

Second, we investigated whether the presence of symptoms was predicted by individual variation in *betweenness centrality*. First, to better understand how *betweenness centrality* varies among individuals in our population, we investigated whether *betweenness centrality* was dependent on sex, age, and transferee status (i.e., being an individual that moved between subgroups). To test for this, we constructed linear models (LMs) with *betweenness centrality* as the response variable and sex (male or female), age (in years), and transferee status (yes or no) as explanatory variables. Shapiro-Wilk tests and diagnostic plots (residuals vs. fitted values and QQ plots) were used to examine assumptions of normality and homogeneity of variances, with no violations of the assumptions found. Since network measures of different individuals extracted from the same network are not independent of each other [98], we employed node-level permutation tests. We permuted the factors of interest 10,000 times, saving the estimates from each permutation. Then, we compared the distribution of permuted data to the estimate derived from the observed data and estimated the two-sided *p*-value to test for significance, with alpha set at 0.05. Backwards selection was used to remove non-significant factors from the model. These statistical analyses were done using the R-package “ANTs” [93]. As the use of node-level permutation tests is currently under debate due to the issue that *p*-values are corrected by node-based permutation tests but effect sizes are not [99], we also ran parametric regression models without permutations. Effect sizes were found to be the same as the permutation models.

Thereafter, we investigated whether *betweenness centrality* could predict the occurrence of symptoms and whether symptoms were associated with sex or age of the individual. Infection was treated as a binary response variable, so we constructed general linear models (GLMs) with a binomial distribution. Sex (male or female), age, and *betweenness centrality* were added as explanatory variables. We also added the interactions between sex and *betweenness centrality* to assess whether social-network position had a different influence on disease risk in males compared to females. Separate models were run, treating age either as a continuous or categorical variable. In our dataset, only one individual was older than 40 years and therefore considered of elderly age [88], making it difficult to treat this as a separate category to test for an effect of elderly age on disease risk. We thus tested juveniles (age 0–6, *n* = 8) versus sub-adults and adults (age 7 and up, *n* = 12). However, as it is currently unclear at what age the immune system of juvenile bonobos is fully developed and thus could be considered an “adult” immune system, we also investigated age as a continuous variable. This analysis revealed highly similar results to the models with age treated as a categorical variable and was therefore not included in the manuscript. Thus, we only report the results from the analysis using age as a categorical variable. Like before, we employed node-level permutation tests, this time permuting the response variable 10,000 times (cf. [57,100]). Parametric regression models were also run, and effect sizes were the same as the effect sizes of the permutation models.

All statistical analyses were done in R [94].

## 3. Results

**Figure 1:**
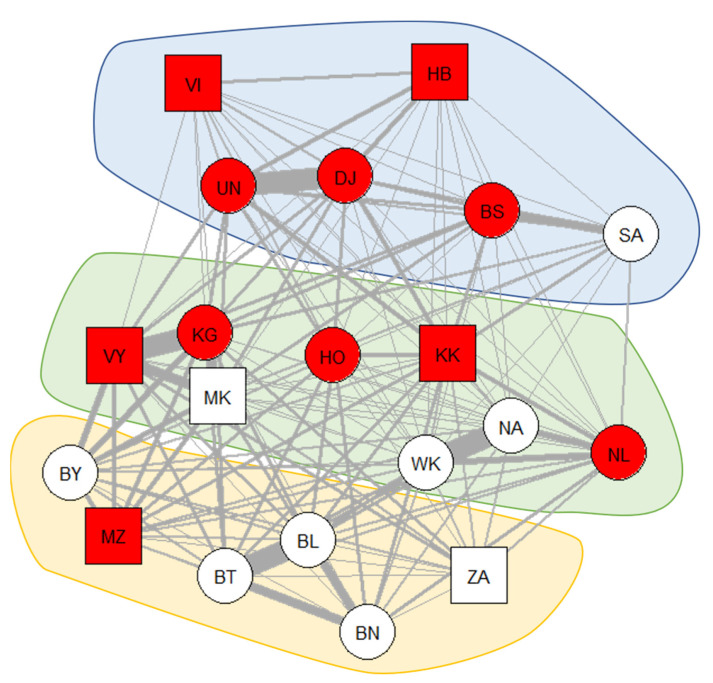
The occurrence of symptoms of respiratory disease in the social-proximity network, including all group members. Red-colored nodes indicate that the individual showed symptoms during the study period, while white-colored nodes represent healthy individuals. Thicker edges indicate that these dyads spend more time in close proximity. Square nodes represent males, while circles represent females. The network was visualized using the Fruchterman-Reingold layout from the “igraph” package [101] in R [94]. Polygons were overlayed based on subgroup membership and transferee status (blue = subgroup 1, no transferee; yellow = subgroup 2, no transferee; green = variable subgroup membership, transferee).

Symptoms of respiratory disease were observed in 11 of the 20 individuals (55%) across the study period. Figure 1 shows the occurrence of symptoms of respiratory disease in the weighted proximity network of the bonobo group.

The path-based k-test indicated that the weighted proximity network containing all individuals (*N* = 20) was epidemiologically relevant to the spread of respiratory disease in the bonobo network. Bonobos that showed symptoms during the study period were closer to each other in the proximity network than expected by chance (mean pathlength to the nearest case: 0.048, *p* = 0.042). The social network without dependent infants (*N* = 16) remained marginally significant (mean pathlength to the nearest case: 0.048, *p* = 0.060).

Subsequently, we investigated which factors were associated with individual variation in *betweenness centrality*. When investigating the whole network, including dependent infants, we found that *betweenness centralities* were not associated with sex (β = −0.794, t = −0.302, *p* = 0.789), age (β = 0.057, t = 0.524, *p* = 0.598), and/or being a “transferee” (i.e., being an individual that switched subgroups during the study; β = 2.321, t = 0.900, *p* = 0.385). The same results were obtained from the smaller network excluding dependent infants (sex: β = −2.314, t = −0.747, *p* = 0.475; age: β = 0.063, t = 0.453, *p* = 0.628; “transferee”: β = 2.075, t = 0.679, *p* = 0.522). An overview of the test statistics can be found in Supplementary Table S3.

**Figure 2:**
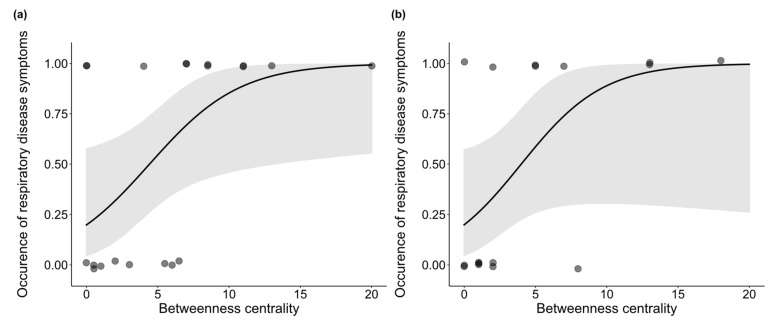
*Betweenness centrality* is positively correlated with occurrence of respiratory disease symptoms both in the (**a**) full network (*p* = 0.002) and (**b**) in the network excluding dependent infants (*p* = 0.023). Shaded area represents the confidence interval.

**Figure 3:**
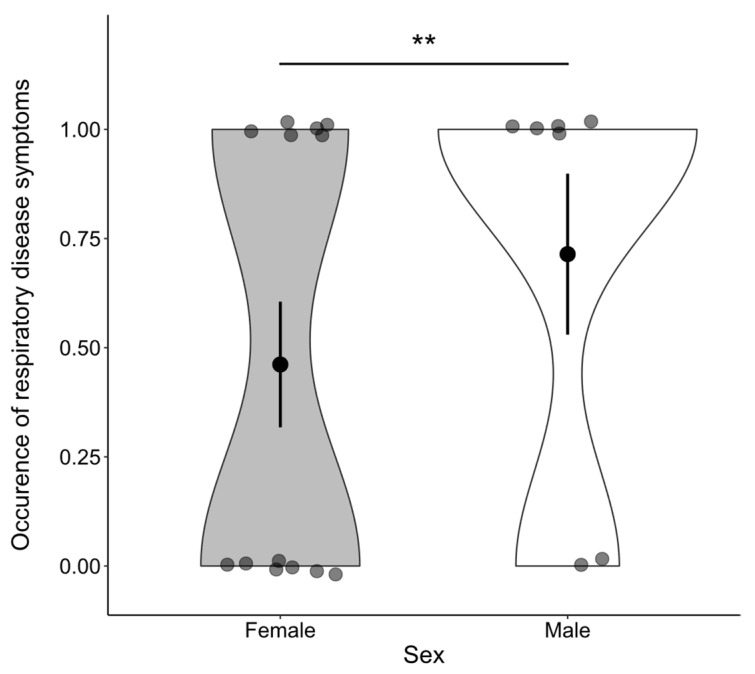
Males had a significantly higher occurrence of respiratory disease symptoms than females (** indicates *p* < 0.01). Error bars indicate 1 SE above and below the mean.

Finally, we investigated the association between the occurrence of symptoms and *betweenness centrality*, sex, and age. Analysis of the whole group network, including dependent infants, revealed that the occurrence of symptoms was positively associated with *betweenness centrality* (β = 0.650, z = 1.922, *p* = 0.002) (Figure 2a) and that males had a higher incidence of symptoms than females (z = 1.714, *p* = 0.007; mean males: 0.714 ± 0.184 SE, mean females: 0.462 ± 0.144 SE) (Figure 3). No significant association between symptom occurrence and age was found (β = −0.556, z = −0.400, *p* = 0.608), nor was there a significant interaction effect between sex and *betweenness centrality* (β = −0.224, z = −0.317, *p* = 0.488). Finally, we retested the effect of *betweenness centrality* on the occurrence of symptoms in a network excluding the dependent infants and found that it is still significant (β = 0.348, z = 1.774, *p* = 0.023) (Figure 2b). An overview of the test statistics can be found in Supplementary Table S4.

## 4. Discussion

In this study, we investigated to what extent social-network position, sex, and age could predict the occurrence of respiratory disease symptoms in a group of zoo-housed bonobos. We found that the proximity network was relevant in explaining the occurrence of respiratory disease, with the pattern remaining marginally significant even when excluding dependent infants. Further analysis showed that in accordance with our predictions, the prevalence of symptoms was higher in males than females and higher in individuals with higher *betweenness centrality*, a measure representing the number of times an individual occurs on the shortest path between two other individuals in the network [24]. This finding was independent of whether dependent infants were included in the network. In contrast to our predictions, no effect of age on disease risk was found.

By conducting a path-based k-test, we found that the proximity network containing all group members was relevant in explaining disease transmission, supporting the notion that close proximity among group members facilitates disease transmission. The proximity network excluding dependent infants remained marginally significant in explaining disease transmission. This could have several causes. First, the size of the network without dependent infants was smaller than the complete network, reducing the sample size and therefore possibly reducing our chances of finding significant results. This is supported by the test statistics, which show that while the effect size remains equal, the *p*-value increases slightly above the 0.05 threshold. Nevertheless, it might be the case that the strong connections between dependent infants and their mothers strongly influence the path-based k-test. After all, this test determines whether the mean distance between infected individuals in the social network is shorter than expected by chance and thus evaluates how “close” two cases of infections are within the network. Therefore, if mother and infant are both infected, their close connection in the network might have a strong influence on the k-test. However, only two of the four mother-infant pairs were infected, suggesting that the close bonds between mothers and infants did not fully drive these results, since the effect of the two infected mother-infant dyads could be outbalanced by the two non-infected mother-infant dyads. Finally, it is possible that dependent infants play an active role in disease spread when they become infected and that dependent infants should be included in the analysis. Young animals, while not necessarily showing a higher chance of respiratory disease infection in our sample, can still carry higher viral loads due to their weaker immune systems [51,58,69,70,71,72]. This, combined with the high likelihood that their mother is also infected, makes it more likely for an infected mother-infant pair to spread disease to individuals in close proximity than for single infected individuals. Nonetheless, larger datasets that include more mother-infant pairs are needed to further investigate the role of infants in disease spread in more detail.

Subsequent analysis showed that the occurrence of symptoms of respiratory disease was more likely in individuals with high *betweenness centrality*, when analyzing the full network, including dependent infants. In this dataset, it is possible that issues of pseudoreplication arise, since mother-infant pairs had highly similar *betweenness centrality* measures and showed the same infection status in our study. However, when excluding dependent infants from the analysis, the same positive effect of *betweenness centrality* was found, which suggests that it is unlikely that the association found between social-network position and symptom occurrence is simply due to pseudoreplication effects. In our study, higher *betweenness centrality* correlated with higher respiratory disease risk, which mirrors findings from other social-network studies in primates and other animal taxa that reported positive correlations between *betweenness centrality* and parasite infection using a variety of social-network types [19,26,27,49,50].

Our results mirror findings from an earlier study on chimpanzees, which also found the social-proximity network to be relevant in explaining respiratory disease occurrence [58]. Moreover, they found a positive association between the occurrence of respiratory disease and social network *strength* [58]. While we tested *betweenness centrality* instead of *strength*, making a direct comparison with our study more difficult, their study showed that *strength* and *betweenness centrality* were correlated, leading them to report only results for *strength*. We chose *betweenness centrality* rather than *strength*, as it is considered a more relevant measure for examining patterns associated with the spread of disease in social-network analysis [95]. On the other hand, another similar recent study in gorillas found no association between *eigenvector centrality* in the proximity and contact network and respiratory disease risk and found the social network to be relevant in predicting disease spread in the early stages of only two out of five respiratory disease outbreaks, suggesting only a small role of social behavior in disease spread [57]. Both cases in which the disease outbreak seemed to follow the social network were in two large groups of gorillas (>30 individuals), while in smaller groups, no associations were found. However, group size is not necessarily the factor explaining this phenomenon, as our study of 20 bonobos and the previous study of 30 male chimpanzees did find an association. Rather, the lack of a correlation in gorillas might be explained by the fact that gorillas do not show fission-fusion dynamics. Hence, they might show lower heterogeneity in proximity among group members than chimpanzees and bonobos do, which causes only small variation in the weights of the network edges among group members. Moreover, gorillas also show less affiliative behavior, such as grooming, than other primate species [102], while these behaviors might be crucial for the spread of disease from one individual to another. Therefore, the relationship between social networks and infection risk might not be universal but is potentially dependent on the social system of the species studied. While the results of our study group might not fully represent patterns found in the wild, as fission-fusion dynamics were artificially managed and wild bonobo populations tend to have larger communities [79,88,89], our study, combined with the findings of the aforementioned studies, might suggest an important role of the social network in determining infection risk in primate species, at least in those living in fission-fusion societies. Nonetheless, more research on different primate species, both in the wild and captivity, is needed to assess the role of social networks in respiratory disease risk.

The occurrence of symptoms was found to be higher in males than females, indicating that males are potentially more prone to infection. While this reflects the general tendency found in vertebrates that the occurrence of parasitism and disease is more common in males than in females [59,60,62,63,64], this raises the question of whether males differ in their social behavior from females [60,68] and have, for example, higher *betweenness centrality*, or whether they are more prone to disease due to differences in immunocompetence [60,65,66,67]. However, there was no significant interaction effect between sex and *betweenness centrality* on the occurrence of symptoms, and additional analysis showed that males, in general, do not have higher *betweenness centrality* scores than females, indicating that higher infection rates in males are more likely due to immunological differences than differences in proximity keeping [68].

Contrary to our expectations, transferees between subgroups did not necessarily have higher *betweenness centrality*, even though transferred individuals, in theory, have more opportunities to form bridges between individuals that would otherwise not be in contact due to the division of the two subgroups. In other words, the transferred individuals are expected to connect different parts of the network, although this appeared to not necessarily be the case in our bonobo group. In order to have high *betweenness centrality*, individuals have to spend a considerable amount of time in close proximity to others, but not all individuals necessarily have the tendency to do so, which might explain this finding. We also did not find any link between age and disease susceptibility or between age and *betweenness centrality*. We expected that disease susceptibility would be higher in young individuals in our dataset, since young individuals have weaker immune systems, as indicated by the higher infection rates found in young animals in a wide variety of taxa [51,58,69,70,71,72]. However, since the strength of the immune system also declines with age in adults [73,74,75,76,77], the relationship between age and disease susceptibility might be complex and U-shaped rather than linear [78]. Unfortunately, the number of elderly individuals in our group was insufficient to test this properly, and our analysis using a categorical distinction between juveniles and adults revealed no association. As currently very little is known about the development of the immune system with age in bonobos, further research is needed to provide a clearer view on the association between age and disease risk in this species.

Finally, our combined findings are interesting with regard to future management recommendations to increase the health and welfare of the species. While it might be advisable to halt fission-fusion dynamics altogether during a disease outbreak, sometimes this is not feasible, for example, due to the breeding management of the group. As individuals with higher *betweenness centrality* are more likely to become infected and thus have a higher potential to spread diseases [17], institutions managing primates in a fission-fusion system could limit the spread of pathogens in the group by closely monitoring the health of individuals with high *betweenness centralities* and avoiding transferring these individuals to other subgroups when symptoms of respiratory disease occur. A limitation of such an approach is that social-network analysis requires a large amount of data [103], so institutes would need to implement routine proximity scans into their daily management until a reliable measure for *betweenness centrality* can be calculated. It also remains unclear how stable this measure is across time in bonobos, so further studies investigating its stability are needed, especially when group composition changes due to transfers of individuals between zoos. Moreover, since males had a higher occurrence of respiratory disease symptoms, transfers of males during outbreaks should be limited in an attempt to prevent infection. Combined, this means that females with low *betweenness centralities* are the preferred candidates for transfers during outbreaks and that their age is likely of lesser importance.

Our study has a few limitations. First, similar to other studies [56,57,58], we based our study purely on the occurrence of symptoms and did not have direct evidence of whether the pathogen that caused the disease in the bonobo group was the same for all individuals. However, it is rather unlikely that different individuals were infected with different pathogens since all individuals showed similar symptoms and large temporal overlap of the occurrence of respiratory disease symptoms. Second, we did not have precise data on exactly when an individual became infected. This type of data would be informative to investigate how respiratory disease spread from one individual to the next throughout the network during the study period. However, this would require invasive sampling of each individual (e.g., through nose swabs) on a regular basis. Moreover, symptoms of respiratory disease usually only show after a few days (i.e., the incubation period), making it difficult to pinpoint the exact time when an individual became infected. Symptoms also typically lasted for a few weeks in our study, offering a long period of potential spread to other group members. Accordingly, our analyses could be repeated on a finer temporal scale, for example, in segments of a few days or weeks, to examine disease spread in more detail. However, this increases the risk that reliability of the social-network measures will be compromised, given that the amount of data in each period would be drastically reduced [103]. Third, we cannot completely rule out that the disease was spread due to unknown environmental factors. During the study period, the bonobo group was kept mainly indoors in one large building, which could facilitate disease spread simply through shared space use. However, our analysis using the path-based k-test showed that the social network significantly explained the spread of infection, indicating that disease spread did not happen randomly. This leads us to believe that the influence of shared space on disease spread was minimal during our study. Still, future studies should try to focus on incorporating information on environmental transmission of disease. Finally, we only focused on social-network position, sex, and age as potential explanatory factors for the occurrence of respiratory disease symptoms, while other behavioral aspects could also influence infection risk, such as individual differences in personality [104]. Indeed, personality has been found to influence infection risk in different animal species (e.g., [105,106,107]), but studies investigating respiratory disease risk remain rare. Therefore, future studies that include larger sample sizes could further explore the association between respiratory disease risk and other behavioral variables, besides social-network position, such as personality, to get a more comprehensive view of factors determining respiratory disease risk in primates.

## 5. Conclusions

Our study confirmed the role of social-network position, as measured by *betweenness centrality*, in determining individual disease risk. Symptoms of respiratory disease were also more likely to occur in males than in females, independent of their social-network position. Combined, these results highlight a joint role of close proximity and sex in increased risk of contracting respiratory disease, two factors that could be taken into account for future welfare management of the species.

## Data Availability

The data presented in this study are available upon reasonable request.

## Supplementary Materials

The following are available online at https://www.mdpi.com/article/10.3390/ani11123597/s1, Table S1: Group composition of the studied bonobo group at Zoo Planckendael. For each individual, the sex, age in years, transferee status, *betweenness centrality* in the two proximity networks, and information on the occurrence of respiratory disease symptoms is given. Individuals that are 7 years or older were considered adults, individuals younger than 7 years were considered juveniles. For the dependent infants (all individuals younger than 2 years old), the mother-infant relationships are indicated by equal superscripts, Table S2: Summary of the managed fission-fusion dynamics of the bonobo group at Zoo Planckendael. The group was divided into two subgroups at all times, which was variable in group composition due to transfers of certain individuals. The individuals that were transferred during the transition of one period to the next are indicated with an asterisk. Individuals that showed symptoms of respiratory disease during a certain period are indicated in red, while white individuals did not show symptoms during that period, Table S3: Test statistics obtained from the LM’s investigating the individual characteristics associated with *betweenness centrality* after running 10,000 permutations, Table S4: Test statistics obtained from the GLMs investigating the influence of *betweenness centrality*, sex, and age on the occurrence of respiratory disease symptoms after running 10,000 permutations. For the network excluding infants, we only tested for the effect of *betweenness centrality*.
