# Supplementary material for: Respiratory Disease Risk of Zoo-Housed Bonobos Is Associated with Sex and Betweenness Centrality in the Proximity Network
Source: Animals (Basel). 2021 Dec 19;11(12):3597. doi: 10.3390/ani11123597 (PMC8698162; doi:10.3390/ani11123597)
Supplement: Supplementary file 1 [file animals-11-03597-s001.zip › animals-1472881-supplementary.pdf]

Supplementary information

Table S1. Group composition of the studied bonobo group at Zoo Planckendael. For each individual, the sex, age in years, transferee status, betweenness centrality in the two proximity networks, and information on the occurrence of respiratory disease symptoms is given. Individuals that are 7 years or older were considered adults, individuals younger than 7 years were considered juveniles. For the dependent infants (all individuals younger than 2 years old), the mother-infant relationships are indicated by equal superscripts.

ID	Sex	Age	Transferee	Betweenness centrality		Showed symptoms?
				Network of whole group	Network excluding infants	
HO	Female	43	Yes	13	7	Yes
BY	Female	31	No	6	2	No
VI	Male	27	No	0	0	Yes
BT[a]	Female	24	No	6.5	8	No
DJ[b]	Female	24	No	11	18	Yes
ZA	Male	23	No	0	0	No
BS	Female	17	No	7	5	Yes
KG[c]	Female	16	Yes	8.5	13	Yes
HB	Male	15	No	0	0	Yes
NA[d]	Female	15	Yes	0.5	1	No
MZ	Male	8	No	7	5	Yes
KK	Male	7	Yes	20	13	Yes
BN	Female	6	No	2	1	No
NL	Female	6	Yes	4	2	Yes
MK	Male	5	Yes	1	1	No
SA	Female	4	No	3	2	No
BL[a]	Female	1	No	5.5	/	No
UN[b]	Female	1	No	11	/	Yes
VY[c]	Male	0 (74 days)	Yes	8.5	/	Yes
WK[d]	Female	0 (14 days)	Yes	0.5	/	No

Table S2. Summary of the managed fission-fusion dynamics of the bonobo group at Zoo Planckendael. The group was divided into two subgroups at all times, which was variable in group composition due to transfers of certain individuals. The individuals that were transferred during the transition of one period to the next are indicated with an asterisk. Individuals that showed symptoms of respiratory disease during a certain period are indicated in red, while white individuals did not show symptoms during that period.

Period 1: 14/01/2021 until 21/02/2021		Period 2: 22/02/2021 until 25/02/2021		Period 3: 26/02/2021 until 02/03/2021		Period 4: 03/03/2021 until 26/03/2021	
**Group 1**	**Group 2**	**Group 1**	**Group 2**	**Group 1**	**Group 2**	**Group 1**	**Group 2**
BS	HO	BS	BL	BS	BL	BS	BL
DJ	BL	DJ	BN	DJ	BN	DJ	BN
HB	BN	HB	BT	HB	BT	HB	BT
KG	BT	HO*	BY	HO	BY	HO	BY
MK	BY	KG	MZ	KK	KG*	KK	KG
NA	KK	KK*	ZA	NA	MK*	SA	MK
NL	MZ	MK		NL	MZ	UN	MZ
SA	ZA	NA		SA	VY*	VI	NA*
UN		NL		UN	ZA		NL*

VI	SA	VI	VY
VY	UN	WK	WK*
WK	VI		ZA
	VY		
	WK		

Table S3. Test statistics obtained from the LM's investigating the individual characteristics associated with betweenness centrality after running 10 000 permutations.

Dataset	Factor	Estimate ± SE	t-value	P-value
Whole network	Sex (ref.: female)	-0.794 ± 2.629	-0.302	0.800
	Age (ref.: adult)	0.057 ± 0.109	0.524	0.611
	Transfer (ref.: yes)	2.321 ± 2.577	0.900	0.389
Network excluding infants	Sex (ref.: female)	-2.314 ± 3.099	-0.747	0.467
	Age (ref.: adult)	0.063 ± 0.140	0.453	0.636
	Transfer (ref.: yes)	2.075 ± 3.057	0.679	0.513

Table S4. Test statistics obtained from the GLMs investigating the influence of betweenness centrality, sex, and age on the occurrence of respiratory disease symptoms after running 10 000 permutations. For the network excluding infants, we only tested for the effect of betweenness centrality.

Dataset	Factor	Estimate ± SE	z-value	p-value
Whole network	*Betweenness centrality*	0.650 ± 0.338	1.922	0.002 **
	Sex (ref. female)	3.993 ± 2.330	1.714	0.014 *
	Age (ref. adult)	-0.556 ± 1.389	-0.400	0.608
	Sex : *Betweenness centrality*	-0.224 ± 0.707	-0.317	0.488
Network excluding infants	*Betweenness centrality*	0.348 ± 0.196	1.774	0.023 *
